# Chitosan Oligosaccharide Promotes Junction Barrier through Modulation of PI3K/AKT and ERK Signaling Intricate Interplay in T84 Cells

**DOI:** 10.3390/polym15071681

**Published:** 2023-03-28

**Authors:** Tahir Mehmood, Rath Pichyangkura, Chatchai Muanprasat

**Affiliations:** 1 Chakri Naruebodindra Medical Institute, Faculty of Medicine Ramathibodi Hospital, Mahidol University, Bang Pla, Bang Phli 10540, Thailand; 2 Department of Biochemistry, Faculty of Science, Chulalongkorn University, Patumwan, Bangkok 10400, Thailand

**Keywords:** chitosan oligosaccharide, wortmannin, SC79, junction barrier, AKT, ERK, diarrhea

## Abstract

Chitosan oligosaccharide (COS) is a breakdown product of chitin, a polymer of N-acetyl-D-glucosamine. COS promotes barrier function in intestinal epithelial cells. However, the exact mechanism of COS-induced barrier function remains unknown. This study was aimed to explore the intricate signaling cascades in the junction barrier induced by COS (100 μg/mL) in human intestinal epithelial cells (T84 cells). COS (100 μg/mL) promoted tight junction assembly and increased transepithelial electrical resistance (TEER). COS inhibited FITC-dextran flux in T84 cell monolayers at 2 h, 4 h, 6 h and 24 h post treatment. In addition, the effect of COS on TEER and FITC-dextran flux was abrogated by pre-incubation of wortmannin (2 μM), an AKT (protein kinase B) inhibitor, at 2 h and 4 h post treatment, indicating that COS-induced tight junction integrity was mediated at least in part by AKT activation. COS-induced TEER was amplified at 24 h and 48 h post treatment by pre-incubation with SC79 (2.5 μM), an AKT activator. Moreover, COS induced inhibition of extracellular signal-regulated kinase (ERK) in T84 cells. Wortmannin and SC79 pre-incubation promoted ERK activation and ERK inhibition, respectively, suggesting that COS-induced ERK inhibition was mediated by AKT. Collectively, this study reveals that COS promotes junction barrier integrity via regulating PI3K/AKT and ERK signaling intricate interplay in T84 cell monolayers. COS may be beneficial in promoting junction barrier in intestinal disorders.

## 1. Introduction

Tight junctions (TJs) or barrier junctions, the barriers between the membranes of two adjacent cells, regulate the transportation of ions and solutes through and within the cells by transcellular and paracellular pathways, respectively [1]. Various pathological conditions such as diarrhea, jaundice and edema are associated with disruption of barrier functions of epithelial and endothelial cells [2]. In addition, colorectal cancers and inflammatory bowel diseases are characterized by loss of the epithelial tight junction integrity, which results in not only loss of cell polarity, but also disturbance of paracellular permeability [3,4]. Epithelial tight junction integrity is also compromised by anti-cancer drugs such as epidermal growth factor receptor (EGFR)–tyrosine kinase inhibitors (TKIs), resulting in the onset of diarrhea [5]. Severe diarrhea in cancer patients leads to treatment suspension or dose reduction, which reduces treatment outcome of the patients [6,7,8]. Hence, it is needed to explore new natural compounds to promote barrier junctions.

Chitosan oligosaccharide (COS), with an average molecular weight (MW) < 10,000 Da, is a breakdown product of chitosan and chitin, a biopolymer of N-acetyl-D-glucosamine abundantly found in exoskeleton of arthropods and cell wall of fungi [9]. COS has higher water solubility and lower viscosity compared with chitosan and chitin. COS is readily absorbed through the intestine and is mainly excreted in the urine [9]. A plethora of scientific evidence has reported physico-chemical and biological properties of COS and its derivatives in various fields including agriculture, biomedical engineering, biotechnology, cosmetics, food processing, nutrition, pharmaceuticals, textiles, water and wastewater treatments. COS has great therapeutic activities such as anti-bacterial, anti-diabetic, and anti-cancer, anti-oxidative, anti-inflammatory, and chemo preventive activities [9,10,11,12,13,14,15]. It has been reported that COS with an MW of ~5000 Da is the most potent polymer promoting junction barriers through adenosine 3′, 5′-cyclic monophosphate (AMP)-activated protein kinase (AMPK)-activation and extracellular Ca^2+^-dependent manners in T84 cell monolayers [16]. AMPK acts as a positive regulator of epithelial tight junction and a negative regulator of cAMP-induced chloride secretion [17]. COS-induced activation of AMPK is arbitrated by calcium-sensing receptor (CaSR)-mediated calcium release from the endoplasmic reticulum [ER] in the intestinal epithelial cells [16]. Recently, it has been reported that COS inhibits interferon γ (IFN-γ)-induced programmed cell death ligand 1 (PD-L1) expression via AMPK activation and signal transducer and activator of transcription 1 (STAT1) inhibition in various tumors [18]. Metformin-modified chitosan (Ch-Met) inhibited overexpressed PD-L1 to inhibit DNA damage repair through selective mitochondria accumulation in tumor cells [19]. Biguanide-modified chitosan (Bi-Ch) has been reported to induce disruption of mitochondrial function and reverse drug resistance by inhibiting MDR-1 expression in tumor cells [20]. Moreover, COS induces chemo-preventive activity through activation of AMPK and inhibition of NF-кB in a mouse model of colorectal cancer [21]. Previously, we have reported the anti-diarrheal activity of COS against afatinib-associated diarrhea in colorectal T84 cancer cells. COS promotes tight junction integrity and prevents afatinib-induced potentiation of cAMP-induced chloride secretion by AMPK activation [17].

AKT, also known as protein kinase B (PKB), has a vital role in regulating metabolism, and cell survival [22]. In intestinal epithelial cells, the tight junction protein is regulated by activation of AKT [22,23]. Mitogen-activated protein kinase (MAPK)–ERK has a crucial role in controlling gene expression, cell differentiation, cell cycle, survival, and apoptosis [24,25]. ERK activation induced by constitutive activation of Ras or Raf disrupts epithelial tight junctions [26]. It has been estabilished that various biological activities mediated by AKT and MAPK/ERK pathways are coordinated and there is crosstalk between these two pathways by different negative and positive feedback loops. Mutations or pathophysiological conditions disrupting the crosstalk between AKT and MAPK/ERK signalings lead to disease development through overactivation of the individual pathways [27]. We previously reported that COS promotes tight junction integrity through activation of AKT and inhibition of ERK pathways [17]. However, the exact intricate interplay mechanism between AKT and ERK signaling cascades mediated by COS in inducing barrier junction integrity remains unclear. Therefore, the present study aims to investigate the mechanism of COS (MW ~5000 Da)-induced AKT activation and ERK inhibition in inducing intestinal barrier junction using a human intestinal epithelial cell line (T84 cells).

## 2. Materials and Methods

### 2.1. Materials

T84 cells obtained from American Type culture Collection (Manassas, VA, USA). DMEM/Ham’s F-12 media, penicillin, streptomycin were purchased from Gibco, Thermofisher Scientific, Waltham, MA, USA). Wortmannin and SC79 were purchased from MedChemExpress (New jersey, USA). MTT was purchased from Bio Basic Inc. (Markham, Canada). DMSO and FITC-dextran (MW 4000 Da) were purchased from Sigma Aldrich (St. Louis, MO, USA). Antibodies such as AKT, p-AKT, ERK42/44, p-ERK42/44 and β-actin were purchased from Cell Signaling Technology (Danvers, MA, USA). All other chemicals used were of at least reagent grade and were purchased commercially.

### 2.2. Preparation of COS

COS was prepared and characterized as previously described [28]. Briefly, one hundred grams of shrimp shell was deproteinized by soaking in 1 N solution of NaOH for 24 h followed by demineralization by soaking in 1 N solution of HCl for 24 h. Lipid-soluble substances and pigments were removed by extraction with 95% ethanol at 75 °C. After that, chitin product was deacetylated in 50% (*w*/*w*) NAOH solution. The chitosan product was then dissolved in acetic acid (1% solution) and subjected to enzymatic hydrolysis by chitinase enzymes. The COS was precipitated with NaOH and washed thoroughly with distilled water (DI) until pH was neutral. The final product was lyophilized and stored at room temperature. Size and degree of deacetylation of COS was determined by gel permeation chromatography and by UV spectroscopic method respectively. COS with the molecular weight of 5000 Da at >90% degree of deacetylation was used in the study.

### 2.3. Cell Culture

T84 cells were cultured in 1:1 Dulbecco’s modified Eagle’s medium (DMEM) and Ham’s F-12 medium supplemented with 10% FBS, 100 U/mL penicillin and 100 mg/mL streptomycin. Cells were maintained at 37 °C with 5% CO_2_/95% O_2_ in humidified atmosphere.

### 2.4. Cell Viability Assays

The cytotoxic activity on T84 cells was determined by MTT assays as previously described [17]. Briefly, 5000 cells were seeded in 96-well cell culture plates and cultured for 24 h at 37 °C. Cells were treated with different concentrations of compounds either alone or in combination for 24 h before incubation with MTT reagent (10 µL of 5 mg/mL) at 37 °C for 4 h. Subsequently, 150 µL of DMSO was added to dissolve formazan crystals and absorbance was measured at 570 nm by the Synergy/neo2 multi-mode reader. The data are presented as percentage of cell viability compared to control.

### 2.5. Measurement of Tight Junction Assembly

The measurement of integrity of epithelial tight junction was determined as previously described [17]. Briefly, T84 cells (5 × 10^5^ cells/support) were seeded on a Snapwell permeable support and cultured for 3–7 days to develop monolayers. Media was changed after every 48 h. To assess monolayer integrity, EVOM2 voltohm meter (World Precision Instruments, Inc., Sarasota, FL, USA) with chopstick electrode set was used to measure TEER across the monolayer. The monolayers with TEER > 1000 Ω.cm^2^ for two consecutive days were used in this experiment. Subsequently, monolayers were exposed with DMEM/ham’s F-12 media supplemented with vehicle, wortamannin (2 µM), SC79 (2.5 µM), COS (100 µg/mL) either alone or in combination. TEER was measured at different time intervals. The data were presented as TEER relative to baseline compared to control.

### 2.6. FITC-Dextran Flux Assay

The measurement of integrity of epithelial tight junction was performed by FITC-dextran flux assay as previously described [17]. T84 cells were seeded on a Transwell permeable support and cultured for 7 days before treatment with vehicle, wortamannin (2 µM), SC79 (2.5 µM), COS (100 µg/mL) either alone or in combination for 2 h, 4 h, 6 h or 24 h. FITC-dextran (MW of 4000 Da) was added into the apical media (1 mg/mL) and, one and a half h later, basolateral media was sampled for the determination of FITC-dextran concentration using the Synergy/neo2 multi-mode reader.

### 2.7. Western Blot Analysis

Proteins extracts for Western blot analysis were prepared as previously described [17]. Briefly, T84 cells treated with different concentrations of wortamannin, SC79, and COS either alone or in combination for 2 h, 4 h, 6 h or 24 h were lysed on ice with RIPA cell lysis reagent supplemented with 1% phosSTOP and protease inhibitors (Roche, Mannheim, Germany). Protein concentrations in cell lysates were determined by Bradford reagent using Lowry method. Twenty-five micrograms of proteins were resolved on 10% sodium dodecyl sulfate-polyacrylamide gel electrophoresis and transferred to nitrocellulose membrane. Membrane was blocked for an hour with 5% (*w*/*v*) nonfat milk. Membranes were then incubated overnight at 4 °C with antibodies to AKT (1:1000), p-AKT (1:1000), ERK42/44 (1:1000), p-ERK42/44 (1:1000) or β actin (1:1000). After washing with TBST, the blots were incubated with horseradish peroxidase-conjugated goat anti-rabbit secondary antibodies for an hour at room temperature. After washing with TBST, signals were detected using ECL plus chemiluminescence kit by Bio-Rad ChemiDocTM Imaging System. Densitometry analysis was performed using ImageJ software and presented in graphical format.

### 2.8. Statistics

Data are presented as mean ± S.D. from three independent experiments and statistically compared with untreated vehicle group and/or compared within treated groups using a repeated measures analysis of variance (ANOVA) followed by Tukey’s post hoc test, by GraphPad Prism software. Furthermore, *p*-values < 0.05 were considered statistically significant. Columns not sharing the same superscript letters are statistically significant.

## 3. Results

### 3.1. Effect of COS, Wortmannin and SC79 on T84 Cell Viability

**Figure 1:**
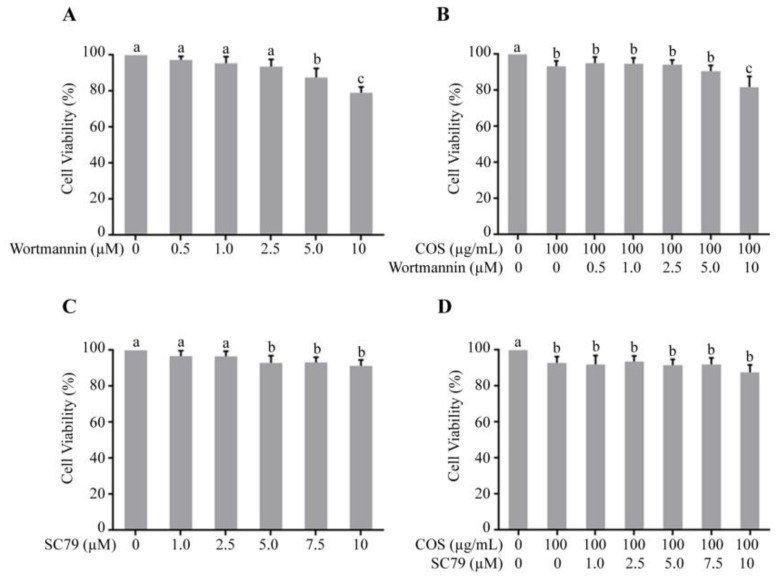
Effect of wortmannin and SC 79 in the presence or absence of COS on T84 cell viability. (**A**) Effect of wortmannin; (**B**) effect of wortmannin combined with COS; (**C**) effect of SC79; (**D**) effect of SC79 combined with COS. Data are expressed as mean ± SD of five independent experiments. Columns not sharing the same superscript letters differ significantly (*p* < 0.05).

Growth inhibitory effect of wortmannin and SC79 in the presence or absence of COS (100 μg/mL) against T84 cells was evaluated by MTT assays. Figure 1A shows that wortmannin did not induce toxicity at concentration up to 2.5 μM and induced statistically significant cell death at 5 μM and 10 μM (Figure 1A). Figure 1B depicted that the effect of COS on cell toxicity was not augmented with pre-incubation with wortmannin. Therefore, a non-toxic concentration (2 μM) of wortmannin was used in subsequent experiments. Likewise, SC79 did not induce toxicity at concentrations up to 2.5 μM and induced statistically significant cell death at 5 μM, 7.5 μM and 10 μM (Figure 1C). The cytotoxic effect of COS was not affected by pre-incubation with SC79 (Figure 1D). Therefore, a non-toxic concentration (2.5 μM) of SC79 was used in subsequent study.

### 3.2. COS Promotes Tight Junction Integrity via AKT Phosphorylation

**Figure 2:**
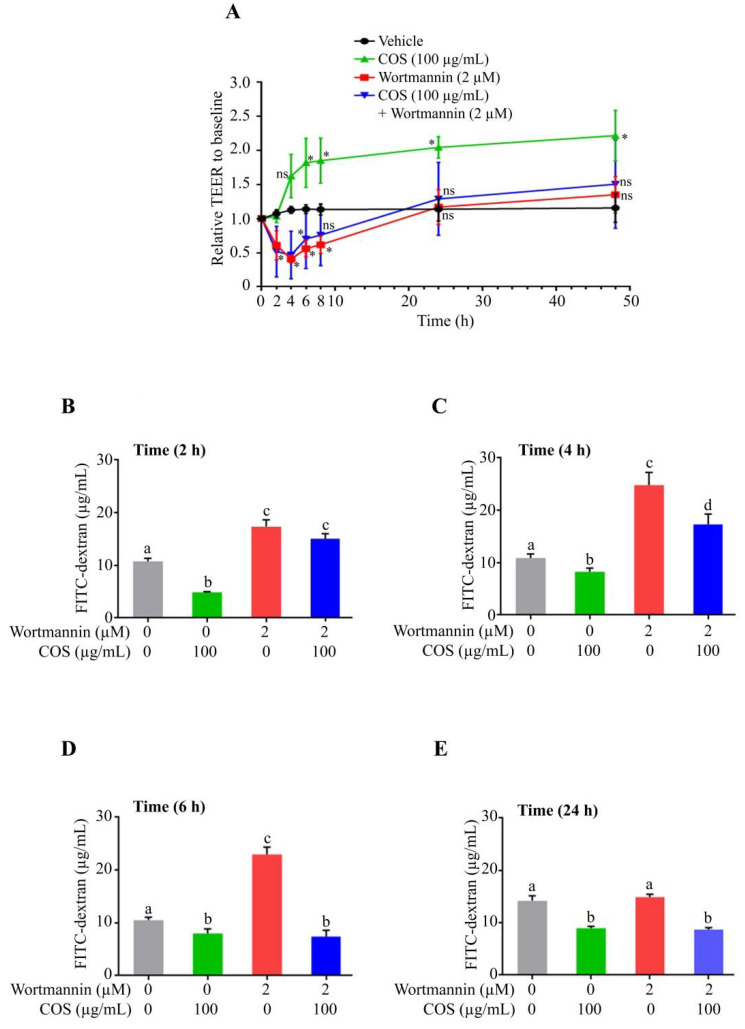
Effect of COS on barrier junction. (**A**) TEER across T84 cell monolayers was measured at indicated time intervals after treatment with indicated concentrations of COS and wortmannin either alone or in combination. Data are expressed as the means of TEER ± S.D. (*n* = 3) (one-way ANOVA; *, *p* < 0.05; ns, non-significant; compared with vehicle treated group). (**B**–**E**) T84 cell monolayers were treated with indicated concentrations of COS and wortmannin either alone or in combination for indicated time intervals followed by FITC-dextran flux assay. Data are expressed as means of FITC-dextran concentration ± S.D. (*n* = 3; one-way ANOVA). Column not sharing the same superscript letter differ significantly (*p* < 0.05).

Previously, it has been reported that COS promotes tight junction assembly through modulation of AMPK, AKT and ERK signaling pathways [16,17]. It has been established that activation of AKT promotes tight junction integrity in intestinal epithelial cells [22,23]. To determine the role of AKT in mediating the tight junction-promoting effect of COS, the effect of COS (100 µg/mL) on tight junction integrity in the presence or absence of wortamannin (2 µM), an AKT inhibitor, was investigated in T84 cells monolayers. As depicted in Figure 2A, COS (100 µg/mL) significantly increased TEER compared to control. Wortmannin (2 µM) reduced TEER significantly at 2 h, 4 h and 6 h post treatment. After 6 h of incubation with wortmannin, TEER started to increase and reached to the level of control group at 24 h and 48 h. The activity of COS (100 µg/mL) on tight junction integrity was eradicated when T84 cell monolayers were pre-incubated with wortmannin (2 µM). These findings suggested that COS-induced tight junction integrity was mediated by activation of AKT. Furthermore, the effect of COS (100 µg/mL) in the presence or absence of wortmannin (2 µM) on tight junction integrity was determined by FITC-dextran (MW of 4 kDa) flux assay. Wortmannin (2 µM) significantly increased FITC-dextran flux at 2 h, 4 h and 6 h post treatment (Figure 2B–D). The effect of wortmannin (2 µM) on FITC-dextran flux was reduced to control level at 24 h treatment (Figure 2E). COS (100 µg/mL) significantly inhibited FITC-dextran flux at 2 h, 4 h, 6 h and 24 h compared to control (Figure 2B–E). COS-induced inhibition of FITC-dextran flux was reversed with pre-incubation with wortmannin at 2 h and 4 h post treatment (Figure 2B,C). With the loss of wortmannin activity at 24 h, COS still significantly inhibited FITC-dextran flux in the presence of wortmannin (Figure 2E), suggesting that the effect of COS on barrier integrity was prolonged by up to 24 h.

**Figure 3:**
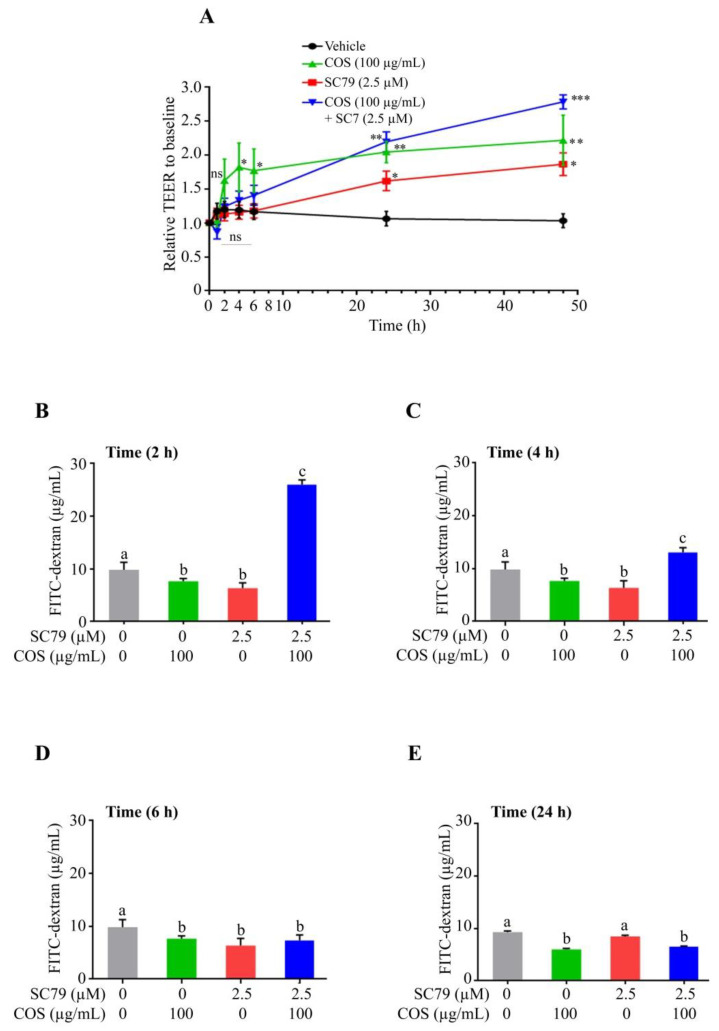
Effect of COS on barrier junction. (**A**) TEER across T84 cell monolayers was measured at indicated time intervals after treatment with indicated concentrations of COS and SC79 either alone or in combination. Data are expressed as the means of TEER ± S.D. (*n* = 3) (one-way ANOVA; *, *p*< 0.05; **, *p* < 0.01; ***, *p* < 0.005; ns, non-significant; compared with vehicle treated group). (**B**–**E**). T84 cell monolayers were treated with indicated concentrations of COS and SC79 either alone or in combination for indicated time intervals followed by FITC-dextran flux assay. Data are expressed as means of FITC-dextran concentration ± S.D. (*n* = 3; one-way ANOVA). Column not sharing the same superscript letter differ significantly (*p* < 0.05, Tukey’s post hoc test).

To further investigate the role of AKT in inducing tight junction integrity, the effect of COS (100 µg/mL) on tight junction integrity in the presence or absence of SC79 (2.5 µM), an AKT activator, in T84 cell monolayers was determined. We found that SC79 (2.5 µM) did not increase TEER until 6 h compared to control, after which SC79 started to increase TEER to an extent significantly higher than that of control at 24 h and 48 h (Figure 3A). When T84 cell monolayers were pre-incubated with SC79 (2.5 µM), the activity of COS (100 µg/mL) on tight junction integrity was lower than that of the group treated with COS alone at 2 h and 4 h. The effect of COS (100 µg/mL) pre-incubated with SC79 (2.5 µM) on TEER was significantly higher than that of COS alone at 48 h (Figure 3A). Likewise, the effect of COS (100 µg/mL) in the presence or absence of SC79 (2.5 µM) on tight junction integrity was determined by FITC-dextran flux assay. SC79 (2.5 µM) significantly decreased FITC-dextran flux at 2 h, 4 h and 6 h post treatment (Figure 3B–D). However, the effect of SC79 (2.5 µM) on FITC-dextran flux was reduced to control level at 24 h post treatment (Figure 3E). COS (100 µg/mL) significantly reduced FITC-dextran flux at 2 h, 4 h, 6 h and 24 h compared to control (Figure 3B–E). Surprisingly, COS-induced inhibition of FITC-dextran flux was reversed with pre-incubation of SC79 (2.5 µM) at 2 h and 4 h (Figure 3B,C), indicating that other signaling events might be initiated with pre-incubation of SC79. Importantly, SC79 pre-treatment did not further increase FITC-dextran flux induced by COS (100 µg/mL) at 6 h and 24 h (Figure 3D,E). These data suggested that COS promoted tight junction assembly by AKT-dependent pathways in T84 cell monolayers.

**Figure 4:**
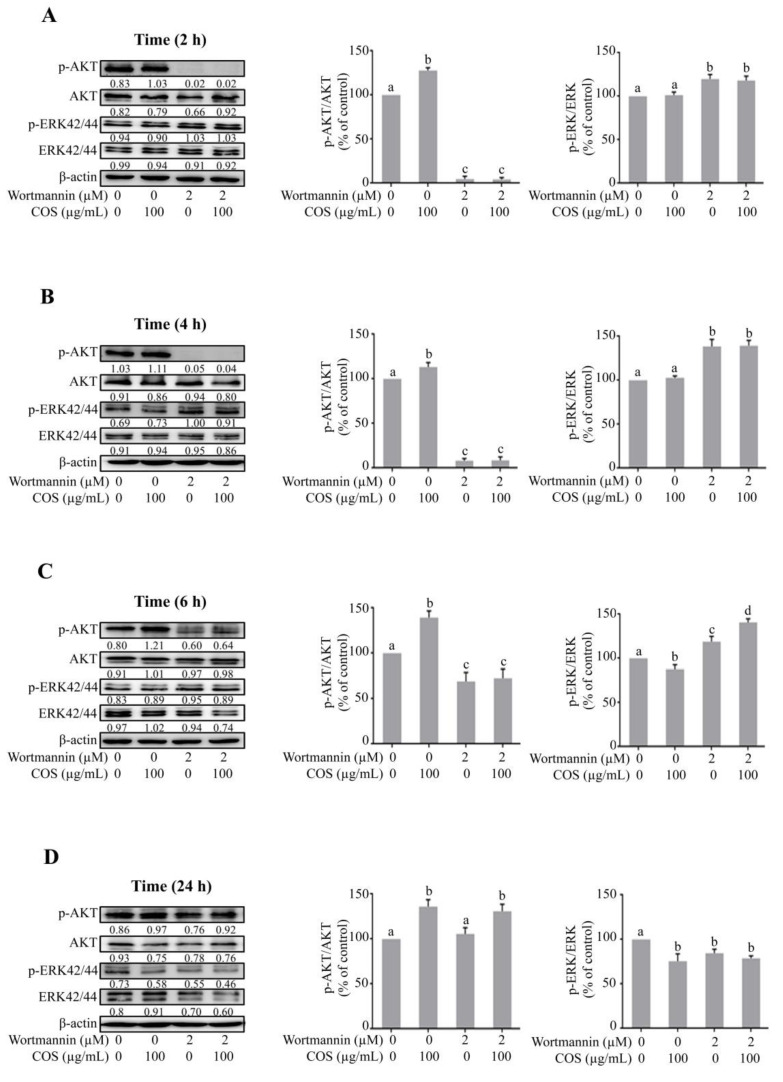
Effect of COS on AKT and ERK phosphorylation studied using wortmannin. (**A**) T84 cells were treated with the indicated concentrations of COS, wortmannin, either alone or in combination for 2 h (**A**), 4 h (**B**), 6 h (**C**), and 24 h (**D**). Data were analyzed as the ratio of p-AKT/AKT, p-ERK/ERK and expressed as % of control (vehicle-treated group), means ± S.D. (*n* = 3). Columns not sharing the same superscript letters differ significantly (*p* < 0.05, one-way ANOVA Tukey’s post hoc test).

Western blot analysis was performed to investigate whether COS induced AKT phosphorylation. It was found that COS (100 µg/mL) significantly induced AKT phosphorylation and increased the ratio of p-AKT/AKT at 2 h, 4 h, 6 h and 24 h (Figure 4A–D). Wortmannin (2 µM) decreased the expression of p-AKT as well as the ratio of p-AKT/AKT at 2 h, 4 h and 6 h (Figure 4A–C) with no significant effect being observed at 24 h post treatment (Figure 4D). The effect of COS (100 µg/mL) on AKT phosphorylation was completely abolished by pre-treatment with wortmannin (2 μM) at 2 h and 4 h and partially suppressed at 6 h post treatment (Figure 4A–C). These results suggested that COS (100 µg/mL) induced AKT activation, which was inhibited by wortmannin (2 μM), in T84 cells.

### 3.3. COS Induces Inhibition of ERK Phosphorylation

**Figure 5:**
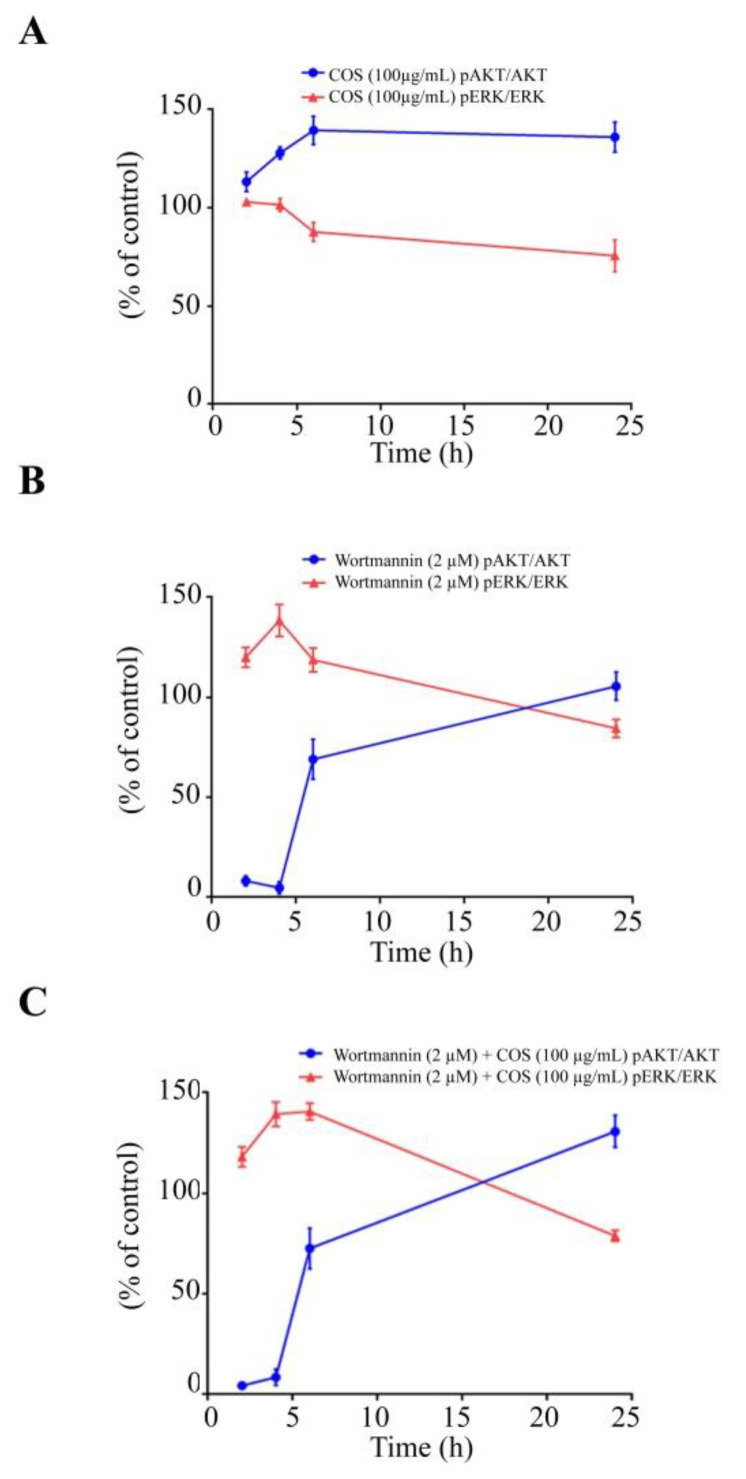
Time course of signaling pathways induced by COS investigated using wortmannin. (**A**) Effect of COS on p-AKT/AKT ratio and p-ERK/ERK ratio. (**B**) Effect of wortmannin on p-AKT/AKT ratio and p-ERK/ERK ratio. (**C**) Effect of COS with wortmannin pre-treatment on p-AKT/AKT ratio and p-ERK/ERK ratio. Data are expressed as % of control (vehicle-treated group) ± S.D. (*n* = 3).

It is known that activation of ERK pathway induces barrier function disruption [26,29]. We next explored the involvement of ERK pathway in COS-induced alteration of tight junction integrity by Western blot analysis. COS (100 µg/mL) did not inhibit ERK phosphorylation and the ratio of p-ERK/ERK at 2 h and 4 h (Figure 4A,B). COS (100 µg/mL) significantly inhibited the expression of p-ERK and the ratio of p-ERK/ERK at 6 h and 24 h (Figure 4C,D). Wortmannin (2 μM) increased the expression of p-ERK and the ratio of p-ERK/ERK at 2 h, 4 h and 6 h (Figure 4A–C), with no effect being observed at 24 h (Figure 4D). COS (100 µg/mL) with wortmannin pre-incubation (2 μM) at 2 h and 4 h post treatment increased the expression of p-ERK and the ratio of p-ERK/ERK, but not significantly different from the wortmannin-treated group (Figure 4A,B). The inhibitory effect of COS (100 µg/mL) on ERK phosphorylation at 6 h post treatment was completely abolished with pre-incubation of wortmannin (Figure 4C). However, as AKT inhibitory activity of wortmannin was lost at 24 h post treatment, COS (100 µg/mL) significantly inhibited ERK phosphorylation and decreased the ratio of p-ERK/ERK at 24 h post treatment (Figure 4D). A summary of the data obtained via Western blot analysis of AKT and ERK phosphorylation is shown in Figure 5. Our data indicated that COS (100 µg/mL) induced activation of AKT through its phosphorylation and increased the ratio of p-AKT/AKT. COS inhibited the activation of ERK and decreased the ratio of p-ERK/ERK. COS-induced increases in p-AKT/AKT ratio were correlated with a decrease in p-ERK/ERK ratio (Figure 5A). Wortmannin inhibited AKT phosphorylation and reversed COS-induced AKT phosphorylation, correlated with increased p-ERK/ERK ratio (Figure 5B,C). These findings suggested that COS-induced tight junction integrity was mediated by activation of AKT and inhibition of ERK signaling pathways.

### 3.4. COS Promotes Tight Junction Integrity via AKT Activation and ERK Inhibition

**Figure 6:**
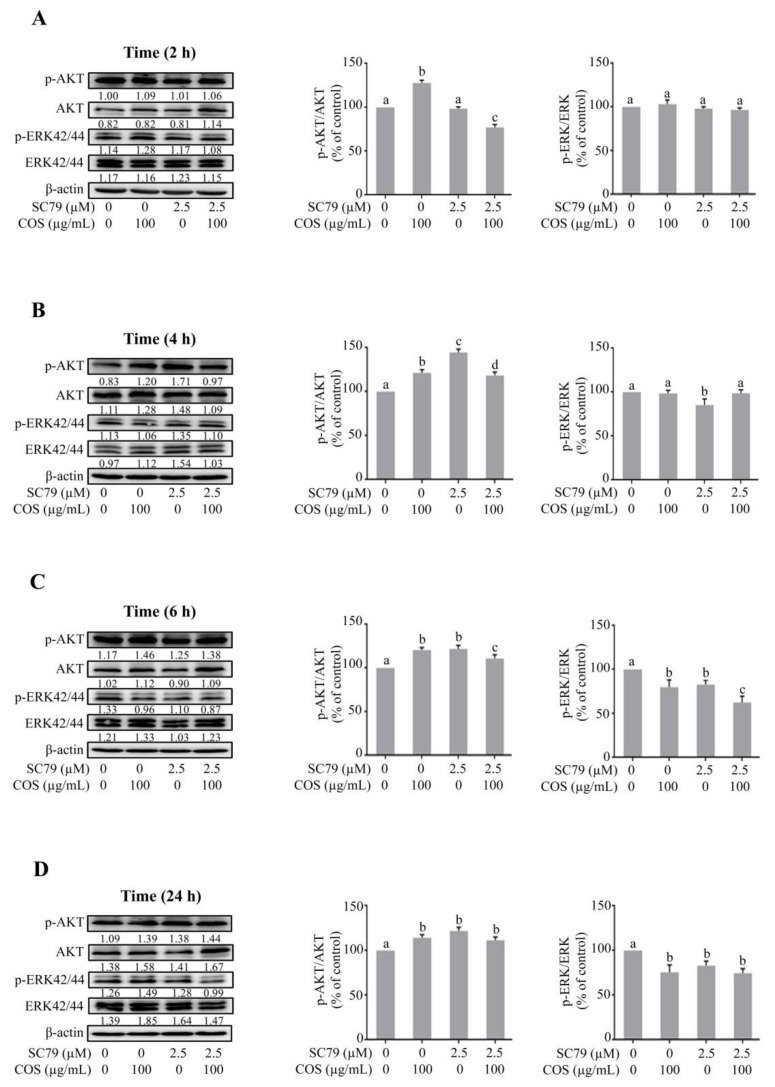
Effect of COS on AKT and ERK phosphorylation studied using SC79. (**A**) T84 cells were treated with the indicated concentrations of COS, SC79, either alone or in combination for 2 h (**A**), 4 h (**B**), 6 h (**C**), or 24 h (**D**). Data were analyzed as the ratio of p-AKT/AKT, p-ERK/ERK and expressed as % of control (vehicle-treated group), means ± S.D. (*n* = 3). Columns not sharing the same superscript letters differ significantly (*p* < 0.05, one-way ANOVA, Tukey’s post hoc test).

To confirm the role of AKT in mediating the effect of COS on ERK inhibition and tight junction integrity, Western blot analysis was performed in the presence or absence of SC79 (2.5 μM), an AKT activator. COS (100 μg/mL) significantly induced AKT phosphorylation and increased the ratio of p-AKT/AKT at 2 h, 4 h, 6 h and 24 h (Figure 6A–D). SC79 (2.5 μM) did not change the ratio of p-AKT/AKT at 2 h compared to control (Figure 6A). SC79 (2.5 μM) significantly increased the expression of p-AKT and p-AKT/AKT ratio at 4 h, 6 h and 24 h (Figure 6B–D). The p-AKT/AKT ratio of cells treated with COS and SC79 was not significantly higher than that with COS or SC79 alone at 4, 6 and 24 h (Figure 6B–D).

**Figure 7:**
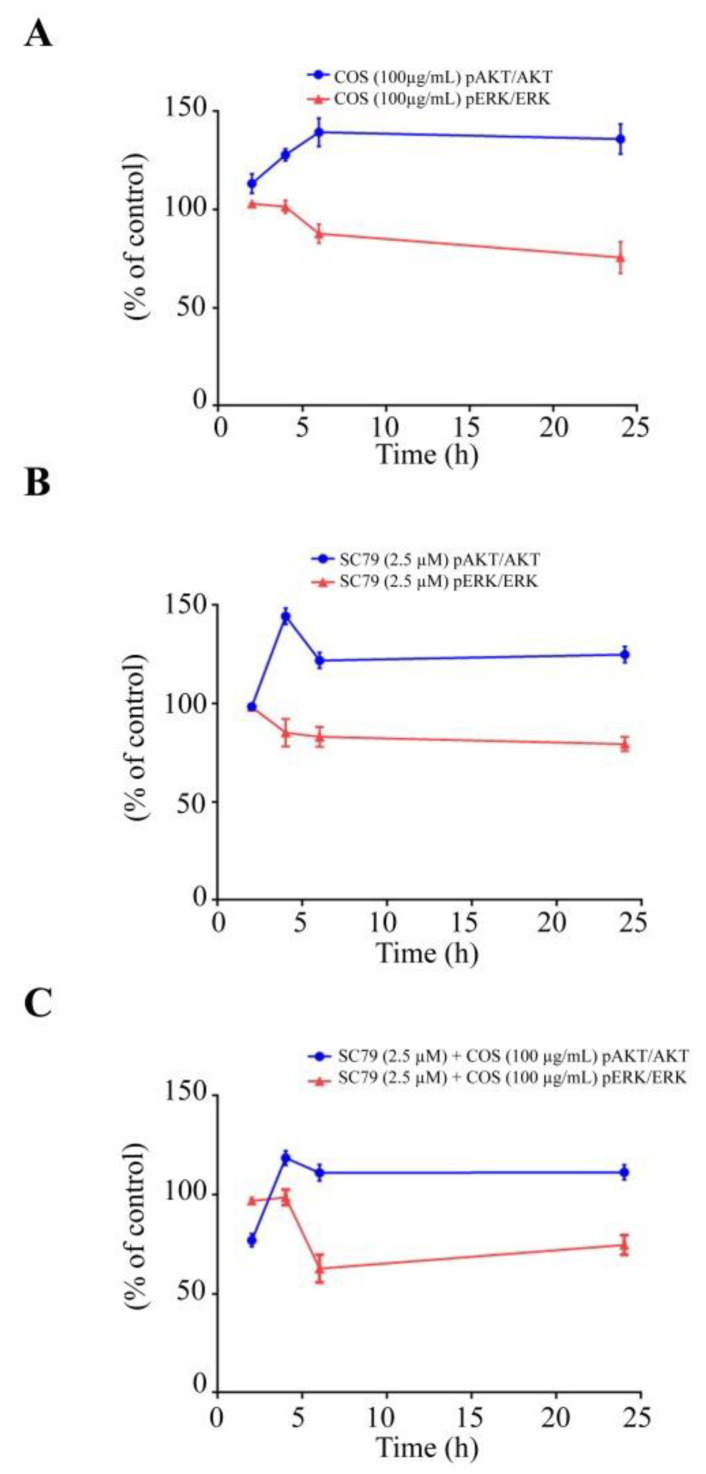
Time course of signaling pathways induced by COS investigated using SC79. (**A**) Effect of COS on p-AKT/AKT ratio and p-ERK/ERK ratio. (**B**) Effect of SC79 on p-AKT/AKT ratio and p-ERK/ERK ratio. (**C**) Effect of COS with SC79 pre-treatment on p-AKT/AKT ratio and p-ERK/ERK ratio. Data are expressed as % of control (vehicle-treated group) ± S.D. (*n* = 3).

We further explored the effect of COS (100 μg/mL)-induced inhibition of ERK pathway in the presence or absence of SC79 (2.5 μM) by Western blot analysis. COS (100 μg/mL) did not induce an inhibitory effect on ERK phosphorylation and the ratio of p-ERK/ERK at 2 and 4 h (Figure 6A,B). COS (100 μg/mL) at 6 h and 24 h significantly inhibited ERK phosphorylation and decreased the ratio of p-ERK/ERK (Figure 6C,D). SC79 (2.5 μM) did not induce any changes in p-ERK expression and the ratio of p-ERK/ERK compare to control at 2 h (Figure 6A). SC79 (2.5 μM) decreased p-ERK expression and the ratio of p-ERK/ERK at 4 h, 6 h and 24 h (Figure 6B–D). The inhibitory effect of COS on ERK phosphorylation and the ratio of p-ERK/ERK was unaffected by pre-incubation with SC79 (2.5 μM) at 2 h and 4 h post treatment (Figure 6A,B). However, the ratio of p-ERK/ERK in cells treated with COS and SC79 was comparable to that with COS or S79 alone (Figure 6C,D). Time course of changes in p-AKT/AKT ratio and p-ERK/ERK ratio in each group is summarized in Figure 7. Our findings suggested that COS- and SC79-induced AKT phosphorylation resulted in inhibition of ERK phosphorylation (Figure 7A,B). SC79 augmented the COS-induced AKT phosphorylation resulting in decreased ERK phosphorylation (Figure 7C).

## 4. Discussion

PI3K/AKT pathway plays a pivotal role in regulating cell metabolism, proliferation and cell survival [22,30,31]. It is established that proteins promoting tight junction are upregulated by activation of AKT in intestinal epithelial cells [22,23]. Our previous report showed that activation of AKT by COS promoted junction integrity in T84 cell monolayer, which is in line with the current report [17]. Furthermore, this study indicates that COS-induced junction barrier was mediated by activation of AKT as pretreatment with SC79, an AKT activator, and wortmannin, an AKT inhibitor, promoted and reversed the effect of COS on TEER and FITC-dextran flux in T84 cell monolayers, respectively.

The MAPK-ERK pathway regulates gene expression, cell differentiation, cell cycle, survival, and apoptosis [25,32,33,34]. Previous studies have shown that ERK activation induced by constitutive activation of Ras or Raf disrupts epithelial tight junctions [26,29]. Our previous report showed that COS inhibited ERK activation in T84 cell monolayer, which is in agreement with the current data indicating that COS-induced barrier junction integrity was mediated by inhibtion of the ERK pathway.

It well established that several biological activities accomplished by AKT and MAPK/ERK pathways are well coordinated, and there is a crosstalk between these two pathways by different negative and positive feedback loops. Any changes in this intricate interplay mechanism between AKT and ERK signaling cascades by mutations or other pathophysiological conditions leads to disease development through over-activation of the individual pathways [35].

AKT is activated by several mechanisms controlled by multiple signaling cascades. AMPK is an essential regulator for AKT activation under stress conditions. AMPK is activated by phosphorylation of AMPK-α subunit at threonine-172 in response to growth factors (GF) through Ca^2+^/calmodulin-dependent protein kinase kinase β (CaMKKβ) and is essential for GF-mediated AKT activation and biological functions [36,37,38]. Previously, we and others have reported that compound C, an AMPK inhibitor, inhibits the activation of AKT indicating that AMPK is an important regulator of AKT pathway [17,39]. Moreover, AKT is also activated by direct activation of epidermal growth factor receptor (EGFr) [40]. In addition, high stimulation of insulin growth factor (IGF) activates AKT through its phosphorylation [27]. Activated AKT inhibits the activity of Raf with concomitant inhibition of ERK phosphorylation [27,35]. Consistent with this notion, there is the possibility of activation of ERK with inhibition of AKT activation. Furthermore, AKT signaling is negatively regulated by ERK activation. Firstly, EGF-induced ERK activation decreases the phosphorylation of Grb2-associated binding protein 1 (Gab1) and inhibits the association of Gab1 with phosphatidylinositol-3-Kinase (PI3K), an upstream of AKT signaling [27]. Secondly, ERK activation by mitogens inhibits liver kinase B1 (LKB1), which is an upstream regulator of AMPK signaling [41]. Consistent with the established notion, inhibition of ERK leads to PI3K/AKT activation [42]. The intricate AKT-ERK interplay might be regulated by types of stimuli such as ligands and concentrations of ligands. Kinetics and spatial factors including co-localization of both AKT and ERK kinases play vital roles in this interplay. Strong stimuli of mitogens quickly activate AKT as Raf activation and recruitment to the plasma membrane occurs slightly later than that of AKT. Activated AKT inhibits Raf with concomitant inhibition of downstream ERK. Mild or weak stimuli may activate AKT not to the extent to inhibit Raf resulting in the activation of ERK [43].

**Figure 8:**
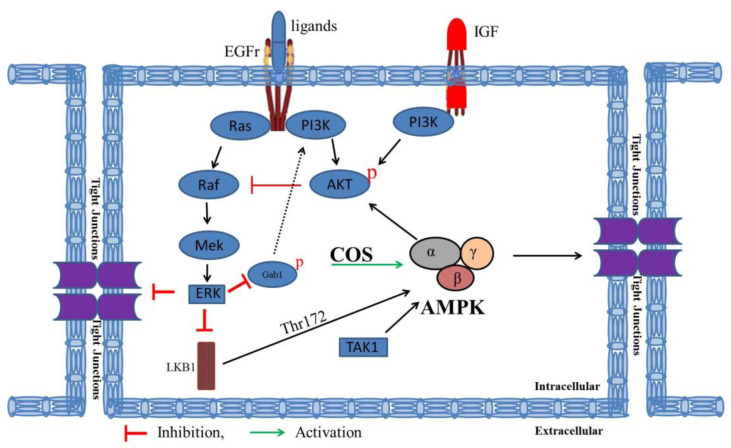
Schematic model summarizing the barrier junction integrity induced by COS via regulating the intricate interplay between PI3K/AKT and MAPK/ERK signaling. AMPK is an essential regulator for AKT activation under stress conditions. AMPK is activated by phosphorylation of AMPK-α subunit at threonine-172 (Thr-172). Moreover, AKT is activated by direct activation of EGFr through ligand binding. High stimulation of insulin growth factor (IGF) also activates AKT through its phosphorylation. Activated AKT inhibits the activity of Raf with concomitant inhibition of ERK. In addition, AKT signaling is negatively regulated by ERK activation. Firstly, EGF-induced ERK activation decreases the phosphorylation of Gab1 and inhibits the association of Gab1 with PI3K, an upstream of AKT signaling. Secondly, ERK activation by mitogens inhibits LKB1, which is an upstream regulator of AMPK signaling.

The data obtained from Western blot analysis were in agreement with the established notion and indicated that COS-induced barrier junction integrity was mediated by activation of AKT through its phosphorylation. Activation of AKT induced by COS inhibited ERK phosphorylation and decreased the ratio of p-ERK/ERK. Pretreatment with wortmannin, an AKT inhibitor, inhibited AKT phosphorylation and reversed COS-induced AKT phosphorylation, which was correlated with the increased p-ERK/ERK ratio. These findings were well supported by studies using SC79, an AKT activator. Pretreatment with SC79 induced AKT phosphorylation and increased COS-induced AKT phosphorylation, correlated with decreased p-ERK/ERK ratio. A schematic model summarizing the barrier junction integrity induced by COS via regulating the intricate interplay between PI3K/AKT and MAPK/ERK signaling is shown in Figure 8.

## 5. Conclusions

This study demonstrates that COS promotes tight junction integrity in T84 cell monolayers. COS-induced tight junction integrity is mediated by activation of AKT and inhibition of ERK signaling cascades. AKT activation induced by COS suppresses ERK activation, which is known to disrupt tight junction integrity [26]. Further studies using in vivo models or other pre-clinical models are needed to investigate efficacy of COS in modulating these multiple pathways for the development of COS as safe and effective therapies against intestinal diseases associated with disrupted barrier function.

## Data Availability

All relevant data are in the manuscript.
